# Worry and ruminative brooding: associations with cognitive and physical health in older adults

**DOI:** 10.3389/fpsyg.2024.1332398

**Published:** 2024-07-03

**Authors:** Rachel M. Morse, Freya Koutsoubelis, Tim Whitfield, Harriet Demnitz-King, Valentin Ourry, Josh Stott, Anne Chocat, Eglantine Ferrand Devouge, Zuzana Walker, Olga Klimecki, Fabienne Collette, Gael Chetelat, Julie Gonneaud, Geraldine Poisnel, Natalie L. Marchant, for the Medit-Ageing Research Group, Florence Allais, Claire André, Eider Arenaza-Urquijo, Romain Bachelet, Sebastian Baez Lugo, Thorsten Barnhofer, Maelle Botton, Nina Coll-Padros, Robin De Flores, Vincent De La Sayette, Marion Delarue, Stéphanie Egret, Hélène Espérou, Eric Frison, Karine Goldet, Idir Hamdidouche, Marc Heidmann, Agathe Joret Philippe, Elizabeth Kuhn, Renaud La Joie, Brigitte Landeau, Gwendoline Le Du, Valérie Lefranc, Maria Leon, Dix Meiberth, Florence Mezenge, Inés Moulinet, Cassandre Palix, Léo Paly, Anne Quillard, Géraldine Rauchs, Stéphane Rehel, Florence Requier, Leslie Reyrolle, Laura Richert, Ana Salinero, Eric Salmon, Raquel Sanchez, Lena Sannemann, Ann-Katrin Schild, Marco Schlosser, Clémence Tomadesso, Edelweiss Touron, Denis Vivien, Patrik Vuilleumier, Cédrick Wallet

**Affiliations:** ^1^Division of Psychiatry, Faculty of Brain Sciences, University College London, London, United Kingdom; ^2^Normandy University, UNICAEN, INSERM, U1237, PhIND “Physiopathology and Imaging of Neurological Disorders”, NeuroPresage Team, Cyceron, Caen, France; ^3^Normandie Univ, UNICAEN, PSL Université, EPHE, INSERM, U1077, CHU de Caen, GIP Cyceron, NIMH, Caen, France; ^4^Department of Clinical, Education and Health Psychology, University College London, London, United Kingdom; ^5^Normandie Univ, UNIROUEN, Department of General Practice, Rouen, France; ^6^Rouen University Hospital, CIC-CRB 1404, Rouen, France; ^7^Essex Partnership University NHS Foundation Trust, Essex, United Kingdom; ^8^Clinical Psychology and Behavioural Neuroscience, Technische Universität Dresden, Dresden, Germany; ^9^GIGA-CRC In Vivo Imaging, Université de Liège, Liège, Belgium

**Keywords:** worry, rumination, cognition, physical health, perseverative cognition, repetitive negative thinking

## Abstract

**Introduction:**

Mental health conditions are associated with cognition and physical function in older adults. We examined whether worry and ruminative brooding, key symptoms of certain mental health conditions, are related to subjective and/or objective measures of cognitive and physical (cardiovascular) health.

**Methods:**

We used baseline data from 282 participants from the SCD-Well and Age-Well trials (178 female; age_mean_ = 71.1 years). We measured worry and ruminative brooding using the Penn State Worry Questionnaire and the Ruminative Response Scale-brooding subscale. We assessed subjective physical health using the WHOQOL-Bref physical subscale, and objective physical health via blood pressure and modified versions of the Framingham Risk Score and Charlson Comorbidity Index. With subjective and objective cognition, we utilized the Cognitive Difficulties Scale and a global composite (modified Preclinical Alzheimer’s Cognitive Composite, PACC5, with the Wechsler Adult Intelligence Scale-IV, category fluency, Mattis Dementia Rating Scale-2, and either the California Verbal Learning Test or the Rey Auditory Verbal Learning Test). We conducted linear regressions, adjusted for education, age, sex and cohort.

**Results:**

Worry and ruminative brooding were negatively associated with subjective physical health (worry: *β* = −0.245, 95%CI −0.357 to −0.133, *p* < 0.001; ruminative brooding: *β* = −0.224, 95%CI −0.334 to −0.113, *p* < 0.001) and subjective cognitive difficulties (worry: *β* = 0.196, 95%CI 0.091 to 0.302, *p* < 0.001; ruminative brooding: *β* = 0.239, 95%CI 0.133 to 0.346, *p* < 0.001). We did not observe associations between worry or ruminative brooding and any measure of objective health.

**Discussion:**

Worry and ruminative brooding may be common mechanisms associated with subjective but not objective health. Alternatively, cognitively unimpaired older adults may become aware of subtle changes not captured by objective measures used in this study. Interventions reducing worry and ruminative brooding may promote subjective physical and cognitive health; however, more research is needed to determine causality of the relationships.

## Introduction

While often considered separate domains of health, there is growing recognition of the interrelationship between cognitive and physical health. As health declines in older adulthood, determining a mechanism(s) that may influence both cognitive and physical aspects will be crucial for developing interventions that promote healthy aging. These facets of health can be investigated using objective measures (e.g., standardized cognitive tests, biomarkers) as well as by measuring subjective perceptions of health. As subjective and objective measures are not always correlated (Cappeliez et al., 2004), using both measures may capture different aspects and contribute to a more comprehensive understanding of health. Additionally, even in the absence of objective health problems, subjective health complaints may represent the beginning of a sequential progression from subtle perception to objective manifestation of worsening objective health. More specifically, in the context of cognitive health, the perception of subjective cognitive decline (SCD), even in the absence of objective impairment, is a risk for subsequent objective cognitive decline and dementia in older adults (Jessen et al., 2014). It is possible that in SCD, concerns arise as individuals become aware of subtle changes in cognition that are not captured by neuropsychological tests (Jessen et al., 2014; Rabin et al., 2017). Similarly, subjective perceptions of physical ill-health in older adults have been shown to predict later morbidity and mortality, even after controlling for objective health measures (Emmelin et al., 2003; van der Linde et al., 2013; Bamia et al., 2017), and have been linked with brain gray matter atrophy (Ourry et al., 2021).

Converging evidence suggests that shared mechanisms may influence both cognitive and physical health such as genetic and psychological factors (Koban et al., 2021). One of the proposed psychological mechanisms, repetitive negative thinking (RNT; also referred to as perseverative cognition), is defined as self-referential, persistent thoughts, that are negative in nature (Ehring and Watkins, 2008). Rumination—past-oriented negative thoughts—and worry—future-oriented negative thoughts—are the key components of RNT (Ehring and Watkins, 2008). Previous studies indicate that there are two divergent components of rumination: ruminative brooding and reflection (Treynor et al., 2003). Ruminative brooding is considered a more maladaptive component of rumination and refers to sullen pondering, whereas reflection is considered a more adaptive component of rumination that focuses on appraisal (Grassia and Gibb, 2008; Armey et al., 2009). Worry is often a key component of generalized anxiety disorder whereas rumination is often a key component of major depressive disorder (Nolen-Hoeksema, 2000; Watkins et al., 2005), individuals with other clinical mental health disorders—as well as non-clinical populations—also engage in worry and rumination (Mansell et al., 2008). These styles of thinking can be experimentally induced, or assessed based on how one is feeling in the present moment, or how one typically feels (i.e., trait). For the purposes of this research, we are particularly interested in trait worry/rumination because, for reasons discussed below, it is the chronic engagement in negative thinking that we believe may have health consequences.

Worry and rumination have been associated with objective and subjective physical and cognitive health in adults of different ages, including increased memory complaints, reduced objective cognitive functioning across several domains including learning and memory, physical health complaints, and pathogenic alterations in the cardiovascular, immune, and endocrine systems (Brosschot et al., 2006; Verkuil et al., 2010; Pietrzak et al., 2012; Ottaviani et al., 2016; de Vito et al., 2019; Marchant et al., 2020; Schlosser et al., 2020). Older adults commonly have poorer physical health (Jette, 1996; El-Gabalawy et al., 2013) and poorer cognitive health (Deary et al., 2009); however, only a limited number of studies have investigated worry and rumination’s associations with health in this population. Of the few studies that have examined worry and/or rumination and cognitive health in older adults, worry has been significantly associated with reduced performance in several domains of objective cognition, including executive functioning and episodic memory (de Vito et al., 2019), and with decline in learning and memory at 2-year follow-up (Pietrzak et al., 2012). An additional study with older adults found that higher levels of RNT were not associated with cognition cross-sectionally but with a faster longitudinal decline in cognition, including global cognition, immediate and delayed memory (Marchant et al., 2020). In relation to subjective cognition, a cross-sectional study found that increased levels of RNT were associated with worse subjective cognition and increased memory complaints (Schlosser et al., 2020). Although these studies provide evidence of a relationship between worry/RNT and poorer objective and subjective cognition, to substantiate these findings, further studies are needed which examine both subjective and objective cognition in the same sample of older adults.

Regarding worry and rumination and physical health in older adults, no studies have investigated worry and only two studies, from the same sample, have investigated trait rumination and physical health in older adults. Thomsen et al. (2004a,b) investigated cross-sectional and longitudinal associations between trait rumination and subjective physical health and immune system activation in younger adults (20–35 years) and older adults (70–85 years). Rumination was positively associated with objective markers of immune system activation and with poorer self-reported subjective physical health in older adults only. When investigated longitudinally, however, higher levels of rumination predicted worsening subjective physical health in younger adults only. These findings highlight age-related differences in relationships between rumination and physical health and call for further studies to investigate and substantiate relationships between rumination and physical health in older adults as well as any relationship between worry and physical health in older adults. Additionally, because experimental induction of worry and rumination impact on cardiovascular response (e.g., blood pressure; Feldman et al., 2004; Key et al., 2008; Ottaviani et al., 2016), it is crucial to understand whether trait levels of worry and rumination exhibit the same relationship in older adults.

Chronic worry and rumination may have widespread effects on the immune, cardiovascular, and endocrine systems in the general population (Brosschot et al., 2006; Verkuil et al., 2010; Ottaviani et al., 2016). This study aimed to examine whether trait-level psychological mechanisms are associated with cognitive and physical health. Though previous studies have focused on rumination generally, this study focused on the more maladaptive component of rumination (e.g., ruminative brooding). Specifically, this study investigated relationships between worry and rumination, independently, and (a) subjective physical health, (b) objective physical health (cardiovascular: Framingham Risk Score, systolic and diastolic blood pressure; comorbidities: Charlson Comorbidity Index), (c) subjective cognitive health, and (d) objective cognition, while controlling for relevant demographic characteristics, in a sample of older adults. We used separate statistical models for worry and rumination to explore whether they would have similar or divergent relationships with these markers of health. We further controlled for symptoms of anxiety and depression to assess whether any observed associations are specific to worry and rumination, respectively.

## Materials and methods

We used baseline data from two randomized controlled trials: SCD-Well (Marchant et al., 2018) and Age-Well (Poisnel et al., 2018). Both trials investigated the effects of behavioral interventions on mental health and wellbeing in older adults. SCD-Well and Age-Well were granted ethical approval by the appropriate ethics committees and were registered on ClinicalTrials.gov (Age-Well: NCT02977819; SCD-Well: NCT03005652). All participants provided written informed consent prior to participation.

### Participants and procedure

*SCD-Well:* SCD-Well included 147 participants recruited from memory clinics at participating study sites (London, United Kingdom; Lyon, France; Cologne, Germany; Barcelona, Spain). Detailed eligibility criteria are provided by Marchant et al. (2018). Briefly, participants were aged 60 years or older and met the research criteria for SCD (i.e., subjectively reported memory decline but performance within a normal range on standardized cognitive tests; Jessen et al., 2014). Clinical anxiety and depression were exclusion criteria, although subthreshold symptoms were permissible.

*Age-Well*: Age-Well included 137 cognitively unimpaired participants recruited from the general population in Caen, France. Eligibility criteria are detailed by Poisnel et al. (2018). Briefly, participants were aged 65 years or older, autonomous and living at home, and performed within the normal range on standardized cognitive tests. Similar to SCD-Well, clinical anxiety and depression were exclusion criteria, although subthreshold symptoms were permissible.

In both cohorts, participants completed a baseline visit where data were collected on medical backgrounds and measures of cognitive, physical, psychoaffective (e.g., anxiety, depression), and biological function (e.g., blood pressure, cholesterol). Both cohorts also collected subjective and objective measures of physical and cognitive health. Nearly all measures were the same for the SCD-Well and Age-Well cohorts; we describe differences in the measures used in further detail below.

### Measures

#### Worry and rumination

We measured trait worry using the Penn State Worry Questionnaire (PSWQ; Meyer et al., 1990). The PSWQ has 16 self-report items on a 5-point scale ranging from 1 (not at all typical of me) to 5 (very typical of me; e.g., “My worries overwhelm me.”). Total scores can range from 16 to 80, with higher scores corresponding to higher levels of worry. The Cronbach’s alpha for the PSWQ is 0.93 (Meyer et al., 1990).

We assessed rumination via the brooding subscale of the Ruminative Response Scale (RRS; Nolen-Hoeksema and Morrow, 1991). The brooding subscale has 5-items on a 4-point scale ranging from 1 (almost never) to 4 (almost always; e.g., How often do you “think ‘Why can’t I handle things better?””). Total scores can range from 5 to 20, with higher scores denoting higher levels of rumination. The RRS has a Cronbach’s alpha of 0.77 (Treynor et al., 2003).

#### Subjective cognition

We used the Cognitive Difficulties Scale (CDS) to measure subjective perception of cognition (McNair and Kahn, 1984). The CDS is a self-report questionnaire containing 39 items measuring subjective ratings of difficulties on a 4-point scale from 1 (never) to 4 (most of the time; e.g., “I forget to return phone calls.”). Items measure difficulties in six domains of cognition: immediate and delayed memory, attention, language, temporal orientation, and psychomotor abilities. Total scores range from 0 to 156, with higher scores indicating worse subjective cognition. The Cronbach’s alpha for the CDS is 0.97 (Gass et al., 2021).

#### Objective cognition

With objective cognition, we used a modified version of the Preclinical Alzheimer’s Cognitive Composite-5 (PACC5; Papp et al., 2017). The PACC5 was designed to measure an track early Alzheimer’s disease-related cognitive decline and encompasses measures of episodic memory, executive function, semantic memory, and global cognition (Donohue et al., 2014). While all PACC5 measures were available in Age-Well, one measure of episodic memory was not available in SCD-Well. As a result, we created a modified PACC5 (referred to as PACC5_Abridged_) in both cohorts. We used the following tests to create the PACC5_Abridged_: the Wechsler Adult Intelligence Scale-IV (WAIS) Coding (raw score), category fluency (total correct), Mattis Dementia Rating Scale-2 (DRS; total score), and either the California Verbal Learning Test (CVLT; delayed free recall) in Age-Well or the Rey Auditory Verbal Learning Test (RAVLT; delayed free recall) in SCD-Well (see Supplementary Table 1 for details).

We computed a PACC5_Abridged_ score for the combined Age-Well and SCD-Well cohorts, by standardizing scores on each of the component measures for all participants with available data and taking the unweighted average of the standardized scores. To investigate the validity of using the PACC5_Abridged_ scores, we created an original PACC5 for the Age-Well cohort using the same method (see Supplementary material for further information). A Pearson’s correlation test comparing the PACC5_Abridged_ scores to the original PACC5 scores in the Age-Well cohort revealed a very strong and significant correlation between the two versions (*r* = 0.96; 95% confidence interval [CI] 0.94 to 0.97; *p* < 0.001).

#### Subjective physical health

We measured subjective physical health using the physical health subscale of the short World Health Organization Quality of Life Measure (WHOQOL-Bref; The WHOQoL-Bref Group, 1995). The subscale includes measures of dependence on medical aids, daily activities, energy levels, fatigue, pain, and discomfort and is comprised of seven items rated on a 5-point Likert scale from 1 (not at all) to 5 (completely; e.g., “How well are you able to get around?”). Total scores range from 7 to 35, with higher scores corresponding to better subjective physical health. The WHOQoL-Bref physical health subscale has a Cronbach’s alpha of 0.82 (Skevington et al., 2004).

#### Objective physical health

We assessed objective physical health with measures of systolic and diastolic blood pressure (SBP, DBP) to examine whether previous associations with induced worry and rumination could be replicated (Feldman et al., 2004; Key et al., 2008; Ottaviani et al., 2016), and because high blood pressure has consistently been associated with a greater risk of kidney disease, heart failure, heart attack, and stroke (Chobanian, 2003).

We also used the Framingham Risk Score (FRS), a 7-item index used to quantify an individual’s 10-year absolute risk of developing coronary heart disease, to measure objective physical health (Assessing Cardiovascular Risk: Systematic Evidence Review from the Risk Assessment Work Group, 2013). Items include ‘yes’ or ‘no’ responses to the presence of behaviors (e.g., smoking) and medications (e.g., blood pressure medication) as well as biological values for cholesterol and blood pressure. The FRS weights items depending on sex and age and sums these with a total score ranging from −10 to 46. Higher scores on the FRS indicate a higher 10-year risk of coronary heart disease and thus, poorer objective physical health.

We calculated an original FRS for Age-Well participants using biological measures of cholesterol and blood pressure and self-report measures for smoking and blood pressure medication. We then calculated an adjusted FRS (referred to as FRS_Adjusted_) for SCD-Well and Age-Well using a medical history questionnaire item where participants responded either ‘yes’ or ‘no’ to having high cholesterol. We scored ‘Yes’ responses 1 and ‘no’ responses as 0 (see Supplementary material for further details). To investigate the validity of the FRS_Adjusted_ as a measure of cardiovascular risk, we ran a Pearson’s rank correlation test comparing the FRS_Adjusted_ to the original FRS in the Age-Well cohort, as the original scores were only available in this cohort. The original and FRS_Adjusted_ were strongly and significantly correlated (*r* = 0.90; 95% CI 0.86 to 0.93; *p* = <0.001).

We also measured objective physical health using the Charlson Comorbity Index (CCI), a 17-item index used to quantify an individual’s burden of diseases and risk of 1-year mortality (Charlson et al., 1987). Items include ‘yes’ or ‘no’ responses to the presence of particular diseases (e.g., peripheral vascular disease) and graded severity responses to the presence of other diseases (e.g., diabetes mellitus: none or diet-controlled, uncomplicated, or end-organ damage; see Supplementary Table 2 for further details). The CCI also includes age as a scored item. Total scores range from 0 to 37 with higher scores corresponding to higher burden of comorbid diseases and higher mortality risk, therefore lower objective physical health.

A medical doctor calculated an original CCI for Age-Well participants during an interview used to determine the presence of CCI conditions. We calculated an adjusted CCI (referred to as CCI_Adjusted_) for both Age-Well and SCD-Well participants as the interview used to create the original CCI was not conducted with SCD-Well participants (see Supplementary material for further information). For the CCI_Adjusted_, we determined the presence of relevant conditions by reviewing (with the support of a medical doctor) participants’ responses to a medical history questionnaire. To investigate the validity of the CCI_Adjusted_ scores as a measure of comorbid disease, we ran a Pearson’s rank correlation test comparing the CCI_Adjusted_ and original CCI scores in the Age-Well cohort. The original and CCI_Adjusted_ scores were significantly correlated (*r* = 0.51; 95% CI 0.36 to 0.63; *p* = <0.001).

#### Psychoaffective measures

We used the Geriatric Depression Scale (GDS) short-form to measure depressive symptomology. The GDS has been specifically designed for use with older adults (Yesavage and Sheikh, 1986). It is a 15-item, self-report questionnaire with ‘yes’ or ‘no’ responses to questions regarding the presence or absence of depressive symptoms (e.g., “Do you often feel helpless?”). Scores range from 0 to 15 with higher scores indicating greater depressive symptomology. The Cronbach alpha for the GDS is 0.75 (Friedman et al., 2005).

We measured anxiety using the 20-item Trait subscale of the State–Trait Anxiety Inventory (STAI-B), a self-report measure of long-standing (i.e., trait) anxiety (Spielberger, 1983). Items are on a 4-point scale from 1 (almost never) to 4 (almost always; e.g., “I feel pleasant”), and the total score ranges from 20 to 80; higher scores indicate higher levels of trait anxiety. For the STAI-B, Cronbach’s alpha is 0.89 (Barnes et al., 2002).

### Statistical analysis

#### Initial analyses

We conducted non-parametric t-tests or chi-square tests for all variables in this study to evaluate any differences between the SCD-Well and Age-Well cohorts.

#### Main analyses

We then ran linear regression models to examine relationships between worry and rumination and physical and cognitive health in the combined Age-Well and SCD-Well cohorts. We ran the analyses with the combined cohorts to increase power to detect relationships. We standardized all variables in the regression models, and conducted linear regressions separately for the outcome variables: WHOQOL-Bref, SBP, DBP, FRS_Adjusted_, CCI_Adjusted_, CDS, and PACC5_Abridged_.

We conducted two linear regression models for each of the outcome variables. In Model 1, we conducted univariate analyses with worry or rumination as the predictor variable. In Model 2, we included the demographic characteristics age, sex, education, and trial (i.e., Age-Well or SCD-Well). The FRS_Adjusted_ includes age and sex in its scoring, thus we only included education and trial in its Model 2. Similarly, the CCI_Adjusted_ includes age in its scoring, thus we only included sex, education, and trial in its Model 2.

#### Sensitivity analyses

We included a number of sensitivity analyses. First, we ran all main analyses separately for each cohort to consider the possibility of having different results for each cohort. Second, we added anxiety (when worry was a predictor) or depression (when rumination was a predictor) as additional covariates, and third, we included worry and rumination in the same model with subjective physical or cognitive health as the outcome variable. Fourth, we ran analyses for the unadjusted CCI_,_ unadjusted FRS, and unadjusted PACC5 in the Age-Well cohort (as these scores were not available in SCD-Well) to consider any differences in results due to the use of adjusted scores in the main analyses. Lastly, we conducted two additional linear regression models for each of the outcome variables with Model A including the demographic characteristics and Model B including the demographic characteristics and worry or rumination.

We conducted all analyses using R version 4.0.2. We set the significance level at *p* < 0.05, and we corrected Model 2 analyses for multiple comparisons (Bonferroni correction *p* < 0.004). These analyses used pairwise deletion to handle missing data. The number of participants included in each part of the analyses is specified with each model.

## Results

We summarized the descriptive statistics for Age-Well (*N* = 135, 84 female; M_age_ = 69.29 years, SD_age_ = 3.75) and SCD-Well (*N* = 147, 95 female; M_age_ = 72.68 years, SD_age_ = 6.87) in Table 1. Participants in SCD-Well were significantly older, had higher levels of rumination and worry, poorer subjective and objective physical health (CCI _Adjusted_ and SBP), poorer subjective and objective cognition (PACC5_Abridged_), and higher levels of anxiety and depression (Table 1). Worry and rumination were significantly correlated in the combined cohorts (*r* = 0.54; 95% CI 0.43 to 0.61; *p* = <0.001).

**Table 1:** Demographic and clinical characteristics of the Age-Well and SCD-Well cohorts.

Variable	Combined cohorts (*N* = 282)	Age-Well (*N* = 135)	SCD-Well (*N* = 147)	*p*-value
Age, years	71.06 (5.85)	69.31 (3.80)	72.68 (6.87)	<0.001^***^
Sex, N (%)				
Female	178 (63.12%)	83 (61.48%)	95 (63.42%)	0.585
Education, years	13.33 (3.44)	13.23 (3.17)	13.43 (3.68)	0.456
Ethnicity, N (%)				
White	-	NR	142 (96.60%)	-
Ruminative brooding				
RRS-brooding^a^	8.44 (2.47)	8.10 (2.29)	8.79 (2.62)	0.037^*^
Worry				
PSWQ^b^	44.11 (12.1)	41.78 (11.55)	46.35 (12.24)	0.002^**^
Subjective physical health				
WHOQoL-Bref^c^	27.65 (4.52)	28.87 (3.65)	26.48 (4.96)	<0.001^***^
Objective physical health				
CCI _Adjusted_^d^	3.69 (1.88)	3.19 (1.65)	4.07 (1.96)	<0.001^***^
FRS _Adjusted_^e^	15.61 (2.82)	15.69 (2.73)	15.53 (2.90)	0.687
SBP^f^	138.32 (21.09)	134.29 (20.70)	141.99 (20.83)	0.005^**^
DBP^e^	79.18 (10.82)	79.61 (10.03)	78.8 (11.5)	0.173
Subjective cognitive health				
CDS^b^	43.33 (20.49)	33.74 (15.06)	52.5 (20.84)	<0.001^***^
Objective cognitive health				
PACC5_Abridged_^e^	0 (1)	0.3 (0.8)	−0.28 (1.09)	<0.001^***^
RAVLT / CVLT_Adjusted_^f^	10.8 (3.33)	11.74 (2.55)	9.94 (3.72)	<0.001^***^
DRS-2^f^	140.4 (3.22)	140.99 (2.65)	139.85 (3.6)	0.006^**^
WAIS-IV^f^	56.52 (13.8)	61.11 (12.45)	52.26 (13.66)	<0.001^***^
Category fluency	31.81 (9.05)	32.27 (8.65)	31.38 (9.41)	0.443
Anxiety and depression				
STAI-B	30.29 (8.29)	27.8 (6.39)	32.58 (9.16)	<0.001^***^
GDS	1.94 (2.15)	1.29 (1.74)	2.54 (2.31)	<0.001^***^

Data are presented as mean (standard deviation) unless otherwise specified. CCI, Charlson Comorbidity Index; CDS, Cognitive Difficulties Scale; CVLT-II, California Verbal Learning Test II; DBP, Diastolic Blood Pressure; DRS-2, Dementia Rating Scale-2; FRS, Framingham Risk Score; GDS, Geriatric Depression Scale; N, Number; NR, not reported; PACC5, Preclinical Alzheimer’s Cognitive Composite 5; PSWQ, Penn State Worry; RAVLT, Rey Auditory Verbal Learning Test; RRS, Rumination Response Scale; SBP, Systolic Blood Pressure; STAI-B, State Trait Anxiety Inventory trait subscale; WAIS-IV, Wechsler Adult Intelligence Scale IV; WHOQoL-Bref, World Health Organization Quality of Life short version. ^a^*N* = 260, ^b^*N* = 276, ^c^*N* = 275, ^d^*N* = 258, ^e^*N* = 280, ^f^*N* = 281.

### Subjective physical health

**Figure 1:**
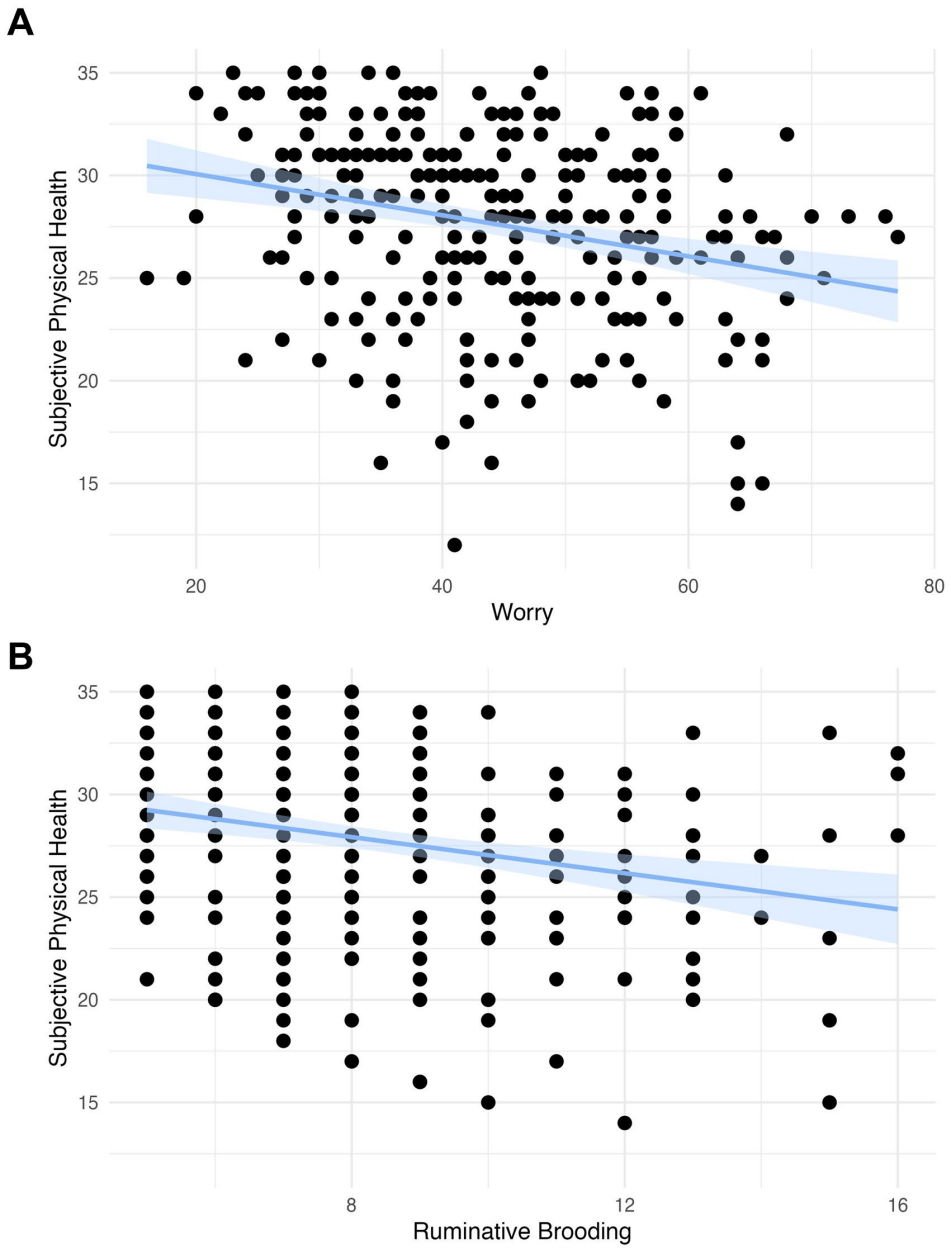
**(A)** The association between worry and subjective physical health with a linear model with a coefficient of −0.440 (scaled: −0.241) and confidence intervals and **(B)** the association between rumination and subjective physical health with a linear model with a coefficient of −0.100 (scaled: −0.268) and confidence intervals.

We observed a significant negative association in both models between worry and subjective physical health (Model 1: *β* = −0.268, 95% CI −0.383 to −0.154, *p* < 0.001; Model 2: *β* = −0.245, 95% CI −0.357 to −0.133, *p* < 0.001) and between rumination and subjective physical health (Model 1: *β* = −0.241, 95% −0.356 to −0.125, *p* < 0.001; Model 2: *β* = −0.224, 95% CI −0.334 to −0.113, *p* < 0.001; Table 2; Figure 1). These findings survived Bonferroni correction for multiple comparisons (*p* < 0.004).

**Table 2:** Associations between worry and ruminative brooding and subjective and objective physical health.

**Subjective physical health**						
WHOQoL-Bref						
	Worry (*N =* 274)			Ruminative brooding (*N =* 258)		
	*Coefficient (95% CI)*	*p-value*	*Adjusted R^2^*	*Coefficient (95% CI)*	*p-value*	*Adjusted R^2^*
Model 1	−0.268 (−0.383 to −0.0154)	<0.001^***^	0.069	−0.241 (−0.356 to −0.125)	<0.001^***^	0.057
Model 2	−0.245 (−0.357 to −0.133)	<0.001^***^	0.169	−0.224 (−0.334 to −0.113)	<0.001^***^	0.167
**Objective physical health**						
CCI_Adjusted_						
	Worry (*N =* 252)			Ruminative brooding (*N* = 236)		
Model 1	0.068 (−0.054 to 0.190)	0.275	0.001	0.044 (−0.085 to 0.173)	0.502	−0.002
Model 2*^a^*	0.024 (−0.098 to 0.147)	0.696	0.041	0.010 (−0.116 to 0.137)	0.875	0.048
FRS_Adjusted_						
	Worry (*N =* 274)			Ruminative brooding (*N* = 258)		
Model 1	0.043 (−0.075 to 0.161)	0.474	−0.002	0.026 (−0.097 to 0.148)	0.683	−0.003
Model 2*^b^*	0.048 (−0.071 to 0.168)	0.428	0.006	0.031 (−0.092 to 0.155)	0.617	0.005
SBP						
	Worry (*N =* 275)			Ruminative brooding (*N =* 259)		
Model 1	0.038 (−0.081 to 0.157)	0.535	−0.002	0.033 (−0.090 to 0.156)	0.599	−0.003
Model 2	0.061 (−0.056 to 0.177)	0.308	0.108	0.042 (−0.076 to 0.161)	0.482	0.104
DBP						
	Worry (*N =* 274)			Ruminative brooding (*N =* 258)		
Model 1	0.039 (−0.079 to 0.158)	0.519	−0.002	0.050 (−0.070 to 0.170)	0.416	−0.001
Model 2	0.039 (−0.083 to 0.161)	0.529	0.008	0.053 (−0.069 to 0.175)	0.397	0.002

CCI, Charlson Comorbidity Index; CI, confidence interval; DBP, Diastolic Blood Pressure; FRS, Framingham Risk Score; N, number; SBP, Systolic Blood Pressure; WHOQoL, World Health Organization Quality of Life short version. Model 1: Unadjusted. Model 2: Adjusted for age, sex, education, and cohort. ^**a**^CCI_Adjusted_ Model 2 includes only sex, education, and cohort. ^**b**^FRS_Adjusted_ Model 2 includes only education and cohort.

### Objective physical health

There was no evidence of a relationship between worry or rumination with any measure of objective physical health (*p*s > 0.05; i.e., SBP, DBP, CCI_Adjusted_, FRS_Adjusted_; Table 2).

### Subjective cognitive health

**Figure 2:**
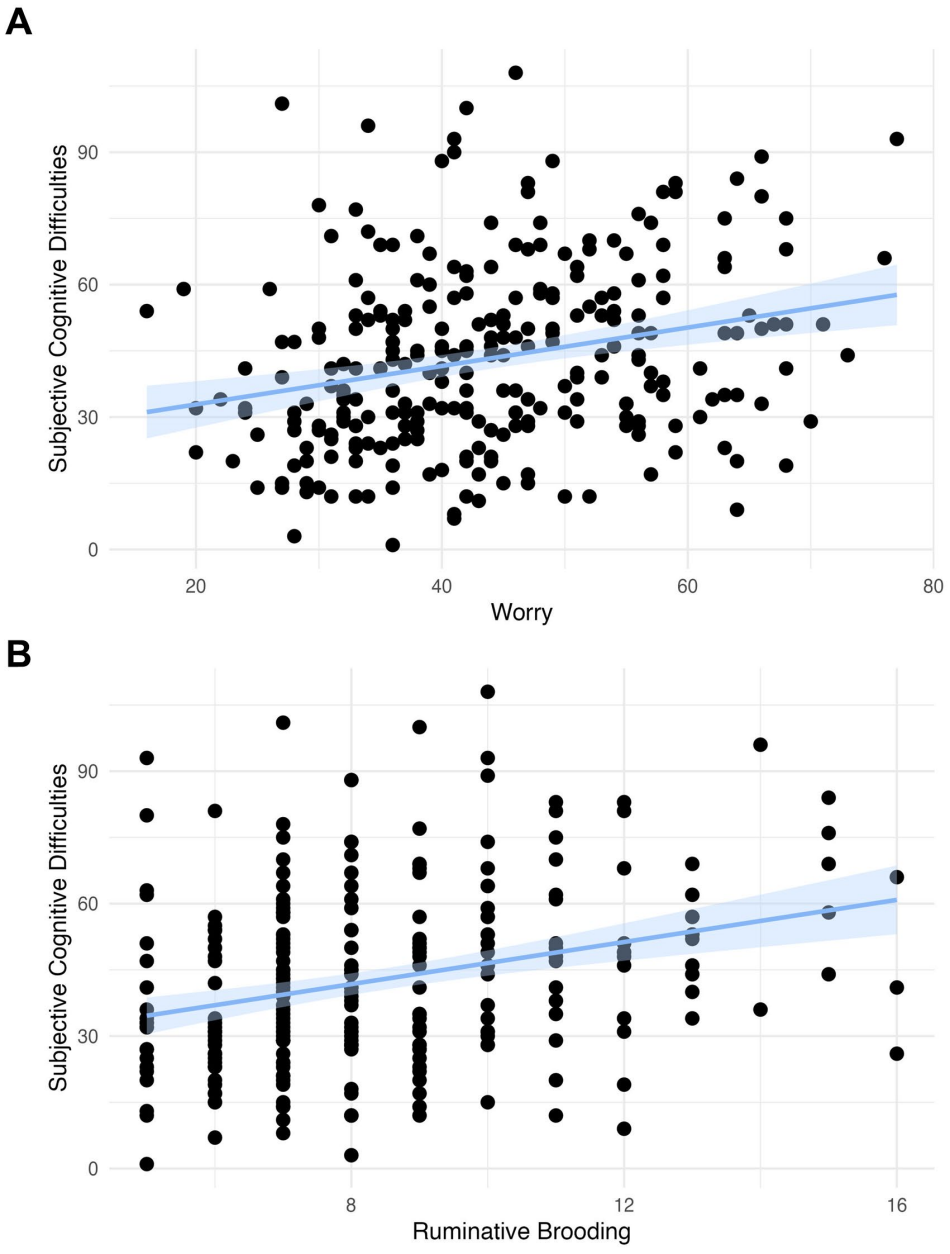
**(A)** The association between worry and subjective cognitive difficulties with a linear model with a coefficient of 0.436 (scaled: 0.257) and confidence intervals and **(B)** the association between rumination and subjective cognitive difficulties with a linear model with a coefficient of 2.386 (scaled: 0.288) and confidence intervals.

We observed a significant positive association in both models between worry and subjective cognitive difficulties (Model 1: *β* = 0.257, 95% 0.142 to 0.372, *p* < 0.001; Model 2: *β* = 0.196, 95% CI 0.091 to 0.302, *p* < 0.001) and between rumination and subjective cognitive difficulties (Model 1: *β* = 0.288, 95% 0.171 to 0.405, *p* < 0.001; Model 2: *β* = 0.239, 95% CI 0.133 to 0.346, *p* < 0.001; Table 3; Figure 2). These findings survived Bonferroni correction for multiple comparisons (*p* < 0.004).

**Table 3:** Associations between worry and ruminative brooding and subjective cognitive difficulties and objective cognitive health.

**Subjective cognitive difficulties**						
CDS						
	Worry (*N =* 275)			Ruminative brooding (*N =* 259)		
	*Coefficient (95% CI)*	*p-value*	*Adjusted R^2^*	*Coefficient (95% CI)*	*p-value*	*Adjusted R^2^*
Model 1	0.257 (0.142 to 0.372)	<0.001^***^	0.063	0.288 (0.171 to 0.405)	<0.001^***^	0.079
Model 2	0.196 (0.091 to 0.302)	<0.001^***^	0.258	0.239 (0.133 to 0.346)	<0.001^***^	0.269
**Objective cognitive health**						
PACC5_Abridged_						
	Worry (*N =* 275)			Ruminative brooding (*N =* 259)		
Model 1	−0.086 (−0.204 to 0.033)	0.157	0.004	−0.081 (−0.206 to 0.043)	0.202	0.002
Model 2	−0.098 (−0.204 to 0.008)	0.071	0.255	−0.079 (−0.188 to 0.031)	0.160	0.245

CDS, Cognitive Difficulties Scale; CI, confidence interval; N, number; PACC5, Preclinical Alzheimer’s Cognitive Composite 5. Model 1: Unadjusted. Model 2: Adjusted for age, sex, education, and cohort.

### Objective cognitive health

There was no evidence of a relationship between worry or rumination and PACC5_Abridged_ scores (*p*s > 0.05; Table 3).

### Sensitivity analyses

Results remained largely unchanged in sensitivity analyses that adjusted for either anxiety or depression (Supplementary Table 3). Further, results also remained unchanged when analyses were conducted in Age-Well and SCD-Well cohorts separately, with the exception of a negative association between worry and objective cognitive health (PACC5_Abridged_) observed in the Age-Well cohort (Supplementary Table 4). The results remained unchanged when using the unadjusted CCI, unadjusted FRS, and unadjusted PACC5 in the Age-Well cohort (Supplementary Table 5); the negative association remained between worry and objective cognitive health (in this case: unadjusted PACC5) in the Age-Well cohort. In analyses with worry and rumination included in the same model, worry and rumination remained significant predictors of subjective physical health and predictors of subjective cognitive difficulties (Supplementary Table 6). Results remained unchanged in sensitivity analyses that included demographic characteristics (Model A) and that included the demographic characteristics and worry or rumination (Model B; Supplementary Tables 7, 8).

## Discussion

This study explored the relationship between worry and rumination and objective and subjective physical and cognitive health in two cohorts of cognitively unimpaired older adults. Worry and rumination were both associated with poorer subjective physical and subjective cognitive health. These relationships remained after adjusting for demographic characteristics and depression or anxiety. There were no significant associations between rumination or worry and any of the objective measures of physical or cognitive health.

The associations between worry/rumination and poorer subjective physical health are in-keeping with previous studies which found that higher levels of rumination and/or worry were associated with an increased number of health complaints in the general population (Rector and Roger, 1996; Lok and Bishop, 1999; Brosschot and van der Doef, 2006; Verkuil et al., 2010) and that higher levels of rumination were associated with poorer self-reported physical health in older adults (Thomsen et al., 2004b). We build on these findings by considering worry and subjective physical health in older adults and including both worry and rumination together in one sample of older adults. Importantly, however, Thomsen et al. (2004b) did not find a relationship between rumination and subjective physical health longitudinally. While the authors suggest this may be due to their sample selection of relatively healthy older adults who may be less susceptible to any effects of rumination, it is also possible that the longitudinal relationships between worry and rumination and subjective physical health differ from the cross-sectional relationships. Further research will need to investigate these measures longitudinally to substantiate the relationships outlined above.

Our findings with subjective cognitive health are in line with an earlier study by Schlosser et al. (2020). They found that worry, rumination, and RNT [measured using the Perseverative Thinking Questionnaire, PTQ (Ehring et al., 2011), a measure of transdiagnostic, content-independent negative thinking] were each associated with greater memory complaints, when considered separately. However, when included in the same model, RNT emerged as the strongest predictor. We have shown that when worry and rumination are included in the same model, both continued to show associations with subjective health, suggesting separate but similar associations with subjective health.

Worry/rumination were not associated with any objective physical or cognitive health measures. Our objective physical health findings contrast with previous literature which found that rumination and/or worry were significantly associated with cardiovascular risk factors, including increased SBP and DBP (Brosschot et al., 2006; Ottaviani et al., 2016; Busch et al., 2017); these discrepancies may result from variation across study designs. The measures of trait rumination and worry included in the present study were designed to capture stable levels of these constructs. Previous studies in the general population have used state measures of worry and rumination intended to capture momentary worry and rumination levels (e.g., Feldman et al., 2004) or used experimental designs that induce worry or rumination and measure immediate effects (e.g., Key et al., 2008). Indeed, Ottaviani et al. (2016) found that higher levels of worry and rumination were associated with increased SBP and DBP in experimental but not observational studies. As such, our results showing a lack of a relationship between worry/rumination and the FRS and blood pressure may be due to our evaluation of long-term rather than transient relationships between these measures.

In relation to the physical comorbidities, there is limited empirical evidence linking worry or rumination with long-term health conditions as assessed in the CCI; however, worry/rumination have been associated with the immune, cardiovascular, and endocrine systems in the general population (Brosschot and van der Doef, 2006; Verkuil et al., 2010; Ottaviani et al., 2016). More specifically, the CCI includes several cardiovascular conditions (e.g., myocardial infarction; Charlson et al., 1987), and worry/rumination have been associated with increased cardiovascular risk factors (Brosschot and van der Doef, 2006; Verkuil et al., 2010; Ottaviani et al., 2016). However, as discussed above, the measures of cardiovascular health may only be associated with worry/rumination in experimental settings. Therefore, worry/rumination may lead to transient effects on physical health systems rather than prolonged effects that could lead to objectively measurable physical health conditions, as assessed in the CCI and FRS.

With objective cognitive health, our null findings are supported by two previous studies showing no relationship between worry (Pietrzak et al., 2012) or RNT (Marchant et al., 2020) and objective cognition cross-sectionally. In both studies, however, relationships emerged over time with worry and RNT predicting greater cognitive decline. A different study by de Vito et al. (2019) did report a negative cross-sectional relationship between worry and objective cognitive health in older adults. Worry levels and variance may have been different in the de Vito et al. study but, given their use of a different worry measure, a direct comparison cannot be made. The de Vito et al. study used a worry assessment designed specifically for older adults (the worry subscale of the Older Adult Self-Report) whereas ours and Pietrzak et al. (2012) used a measure for the general population; therefore, in addition to potential cohort differences, their measure may have been more sensitive/specific to detecting cognitive differences.

The evidence of an association between worry/rumination and subjective but not objective health in older adults may result from differences in the sensitivity of these measures. As participants in the Age-Well and SCD-Well cohorts were older adults without significant impairment, the associations between worry/rumination and subjective health may reflect their awareness of subtle changes in their physical and cognitive health which are not yet captured by objective physical and cognitive health measures. This is supported by evidence that older adults with subjective memory concerns and/or SCD are at a higher risk of objective cognitive decline and dementia (Jessen et al., 2014; Rabin et al., 2017; Hallam et al., 2022) and that poorer subjective health in older adults predicts higher incidence of physical ill-health including cardiovascular health, stroke, inflammation, and stress reactivity (Emmelin et al., 2003; Demakakos et al., 2007; van der Linde et al., 2013; Stephan et al., 2015; Shrira et al., 2016). Better subjective physical health has been associated with greater gray matter volume and white matter microstructural integrity in brains of older adults (Ourry et al., 2021). Alternatively, individuals with higher levels of worry and rumination may be more likely to report poorer outcomes for all subjective measures, irrespective of their objective prognosis due to hyper-attentiveness to even small changes. In this case, poorer subjective health may not precede worsening objective health but instead may reflect a proneness to concern or distress.

It is important to note that the subjective and objective physical health measures in this study measured different aspects of physical health, with the WHOQOL focusing on physical health as it relates to daily functioning (e.g., ability to do activities) rather than their perception of having specific risk factors or illnesses (having high blood pressure, for example). Indeed, Cappeliez et al. (2004) found that subjective physical health accounted for a small proportion of the variance in objective physical health in older adults. These varied representations of subjective and objective physical health may explain the different results observed between subjective and objective physical health in this study.

Strengths of this study include the assessment of worry and rumination in the same sample, which allowed us to determine their distinct and overlapping relationships with markers of health. The fact that their associations were consistent across all analyses supports the shift toward a transdiagnostic approach to the assessment of thinking styles (e.g., the PTQ; Ehring and Watkins, 2008). We further investigated both objective and subjective physical and cognitive health in the same sample, outlining key differences in these representations of health and emphasizing that subjective and objective health can diverge and represent different measures of health. This highlights the importance of including subjective as well as objective ratings in assessments to gain a richer understanding of physical and cognitive health (Stephan et al., 2018). Additionally, this study adjusted for multiple comparisons and potential confounds including demographic characteristics and included a large sample of older adults from two independent cohorts which increased the power to detect smaller effects. Moreover, findings remained largely consistent when the cohorts were examined separately.

The cross-sectional design is one limitation of this study because we cannot infer causality from the results. While we suggest that higher levels of worry and rumination precede poorer subjective physical and cognitive health, poorer subjective physical and cognitive health may precede higher levels of worry and rumination. Future studies examining longitudinal data may elucidate relationships with objective health that might emerge over time. Additionally, participants in this study were highly educated and relatively healthy (physically and cognitively); therefore, the results may not be generalizable to the wider older adult population. This study also uses a variety of self-report questionnaires, which may be influenced by participants’ transient psychological states or response biases. Lastly, we used adjusted FRS, CCI, and PACC5 measures. It is possible that analyses with the original FRS, CCI, and PACC5 would have yielded different results; however, the adapted versions were either strongly (for the FRS and PACC5) or moderately (for the CCI) correlated with originals, and the results remained unchanged when the analyses were conducted in the Age-Well cohort with the original scores.

The associations between worry/rumination and subjective physical and cognitive health in older adults have wider implications for promoting wellbeing in this population. Interventions that target worry or rumination may improve subjective health and in turn psychological wellbeing. For example, an intervention aiming to reduce worry levels in young adults found that participants with reduced worry levels reported better subjective physical health (Brosschot and van der Doef, 2006). Cognitive-behavioral therapy (Watkins et al., 2011) and mindfulness-based meditation (Gu et al., 2015) are behavioral interventions that have been shown to reduce levels of RNT, and also psychological wellbeing (Chételat et al., 2022) In sum, while further research is needed to investigate causality in the relationship between RNT and subjective physical and cognitive health in older adults, interventions aiming to reduce worry and rumination levels in older adults may address mechanisms underlying poorer subjective physical and cognitive health.

## Data availability statement

The data can be made available on request following a formal data sharing agreement and approval by the consortium and executive committee (https://silversantestudy.eu/2020/09/25/data-sharing). The Material can be mobilized, under the conditions and modalities defined in the Medit-Ageing Charter, by any research team belonging to an Academic institution for carrying out a scientific research project relating to the scientific theme of mental health and well-being in older people. Data sharing policies described in the Medit-Ageing Charter are in compliance with our ethics approval and guidelines from our funding body. All analysis code is available (https://github.com/rachmorse/Worry-Rumination-and-Older-Adult-Health).

## Ethics statement

The necessary ethics committees and regulatory agencies approved the SCD-Well trial in London, UK (Queen Square Research Ethics Committee and Health Research Authority), Lyon, France (Comité de Protection des Personnes CPP Sud-Est II Groupement Hospitalier and Agence Nationale de Sécurité du Médicament et des Produits de Sante), Cologne, Germany (Ethikkommission der Medizinischen Fakultät der Universität zu Köln), and Barcelona, Spain (Comite Etico de Investigacion Clinica del Hospital Clinic de Barcelona), and the Age-Well trial in Caen, France (Comité de Protection des Personnes CPP Nord-Ouest III). The studies were conducted in accordance with the local legislation and institutional requirements. The participants provided their written, informed consent to participate in this study.

## Author contributions

RM: Formal analysis, Methodology, Visualization, Writing – original draft, Writing – review & editing. FK: Formal analysis, Methodology, Writing – original draft, Writing – review & editing. TW: Data curation, Formal analysis, Methodology, Supervision, Writing – review & editing. HD-K: Conceptualization, Data curation, Formal analysis, Methodology, Supervision, Writing – review & editing. VO: Data curation, Writing – review & editing. JS: Writing – review & editing. AC: Data curation, Writing – review & editing. ED: Data curation, Writing – review & editing. ZW: Data curation, Writing – review & editing. OK: Writing – review & editing, Funding acquisition. FC: Writing – review & editing, Funding acquisition. GC: Writing – review & editing, Funding acquisition, Resources. JG: Writing – review & editing, Data curation, Investigation. GP: Writing – review & editing, Data curation, Funding acquisition, Project administration. NM: Conceptualization, Data curation, Formal analysis, Funding acquisition, Methodology, Supervision, Writing – review & editing.

## Group members of the Medit-ageing research group

The Medit-Ageing Research Group includes Florence Allais, Claire André, Eider Arenaza-Urquijo, Romain Bachelet, Sebastian Baez Lugo, Thorsten Barnhofer, Maelle Botton, Nina Coll-Padros, Robin De Flores, Vincent De La Sayette, Marion Delarue, Stéphanie Egret, Hélène Espérou, Eric Frison, Karine Goldet, Idir Hamdidouche, Marc Heidmann, Agathe Joret Philippe, Elizabeth Kuhn, Renaud La Joie, Brigitte Landeau, Gwendoline Le Du, Valérie Lefranc, Maria Leon, Dix Meiberth, Florence Mezenge, Inés Moulinet, Cassandre Palix, Léo Paly, Anne Quillard, Géraldine Rauchs, Stéphane Rehel, Florence Requier, Leslie Reyrolle, Laura Richert, Ana Salinero, Eric Salmon, Raquel Sanchez, Lena Sannemann, Ann-Katrin Schild, Marco Schlosser, Clémence Tomadesso, Edelweiss Touron, Denis Vivien, Patrik Vuilleumier, and Cédrick Wallet. Many people helped in implementing this study.
