# Supplementary material for: Worry and ruminative brooding: associations with cognitive and physical health in older adults
Source: Front Psychol. 2024 Jul 3;15:1332398. doi: 10.3389/fpsyg.2024.1332398 (PMC11252046; doi:10.3389/fpsyg.2024.1332398)
Supplement: Supplementary file 1 [file Data_Sheet_1.docx]

**Supplementary Materials**

**CCI**

To create the CCI_Adjusted_, we used a self-report medical history questionnaire to determine the presence or absence of CCI conditions. The medical history questionnaire has ‘yes’ or ‘no’ responses for some of the conditions on the CCI and a write-in section where participants were asked to list all other conditions they have. With the help of a medical doctor, we reviewed all write-in conditions and sorted them into the CCI condition categories, if applicable. It was assumed that if a participant did not list a condition, they did not have the condition. We made additional adjustments to the graded severity responses in the CCI_Adjusted_ when the appropriate data were not available (e.g., diabetes scores originally ranged from 0 (none or diet-controlled) to 2 (end organ damage) while adjusted scores ranged from 0 (no) to 1 (yes)). With particular diseases, we substituted other diseases if deemed appropriate by the medical doctor (e.g., ‘Connective tissue disease’ from the original score was substituted by ‘Rheumatoid Arthritis or other Collagen Vascular disease’ in the adjusted score). Supplementary Table 2 outlines all details of the definitions and scores used in the original CCI and the changes made to create the CCI_Adjusted_.

**FRS**

SCD-Well participants did not have biological cholesterol data collected and instead responded ‘yes’ or ‘no’ to a medical history questionnaire item asking participants whether they have high cholesterol. In the original FRS, HDL and total cholesterol scores ranged between -1 and 4 for participants over the age of 60 years. However, in the FRS_Adjusted_, we used a range for cholesterol levels of 0 to 1 with 0 corresponding to ‘no’ and 1 corresponding to ‘yes’ on the medical history questionnaire item regarding high cholesterol. The corresponding range of total FRS_Adjusted_ scores was 0 to 43. In both the adjusted and original FRS, we scored all participants over the age of 79 years according to FRS quantified risk for the age group 70-79 years as the FRS does not include quantified risk for those older than 79 years.

**PACC5**

SCD-Well participants did not have data from two episodic memory tests available, and as such we created a PACC5_Abridged_. We summarized the measures used in each cohort in the PACC5_Abridged_ in Supplementary Table 1. As the CVLT-II was used in Age-Well and the RAVLT in SCD-Well, we divided the CVLT-II scores by 16 (the number of words to remember in the CVLT-II) and multiplied by 15 (the number of words to remember in the RAVLT) to create a comparable measure of episodic memory. We then standardized the scores for each of the component measures for all participants with available data. We then used the unweighted average of the standardized scores in the combined cohort as the PACC5_Abridged_ for the combined cohorts.

**Supplementary** **Table 1**

*Neuropsychological tests used for the adjusted Preclinical Alzheimer’s Cognitive*

*Composite 5 in Age-Well and SCD-Well*

|  | **Age-Well** | **SCD-Well** |
| --- | --- | --- |
| Global memory | DRS-2 (total score [0-144 points]) | DRS-2 (total score [0-144 points]) |
| Executive function | WAIS-IV Coding (raw score [0-93 symbols]) | WAIS-IV Coding (raw score [0-93 symbols]) |
| Episodic memory | CVLT-II (delayed recall score [0-16 words]) | RAVLT (delayed recall score [0-15 words]) |
| Semantic memory | Category Fluency (1 x 2 minute [number of correct animals stated]) | Category Fluency (1 x 2 minute [number of correct animals stated]) |

Abbreviations: CVLT-II California Verbal Learning Test II, DRS-2 Dementia Rating Scale-2, RAVLT Rey Auditory Verbal Learning Test, WAIS-IV Wechsler Adult Intelligence Scale IV

**Supplementary Table 2**

*Details of the scoring used for the original and adjusted Charlson Comorbidity Index*

| **Original CCI** | | **Adjusted CCI** | |
| --- | --- | --- | --- |
| Myocardial infarction | No (0), Yes (1) | Myocardial infarction | No (0), Yes (1) |
| Congestive heart failure | No (0), Yes (1) | Heart disease | No (0), Yes (1) |
| Peripheral vascular disease | No (0), Yes (1) | Peripheral vascular disease | No (0), Yes (1) |
| Cerebrovascular accident or transient ischemic attack | No (0), Yes (1) | Stroke or transient ischemic attack | No (0), Yes (1) |
| Dementia | No (0), Yes (1) | Dementia | No (0), Yes (1) |
| COPD | No (0), Yes (1) | COPD, asthma, respiratory illness | No (0), Yes (1) |
| Connective tissue disease | No (0), Yes (1) | Rheumatoid Arthritis or other Collagen Vascular disease | No (0), Yes (1) |
| Peptic ulcer disease | No (0), Yes (1) | Peptic ulcer disease | No (0), Yes (1) |
| Liver disease | None (0), mild (1), moderate/severe (3) | Renal insufficiency | No (0), Yes (1) |
| Diabetes | None/diet-controlled (0), uncomplicated (1), end organ damage (2) | Diabetes | No (0), Yes (1) |
| Hemiplegia | No (0), Yes (2) | Hemiplegia | No (0), Yes (2) |
| Moderate/ severe chronic kidney disease | No (0), Yes (2) | Hepatic insufficiency | No (0), Yes (2) |
| Solid tumor | None (0), localized (2), metastatic (6) | Solid tumor | None (0), localized (2), metastatic (6)^*^ |
| Leukemia | No (0), Yes (2) | Leukemia | No (0), Yes (2) |
| Lymphoma | No (0), Yes (2) | Lymphoma | No (0), Yes (2) |
| AIDS | No (0), Yes (6) | AIDS | No (0), Yes (6) |

Note. Abbreviations: CCI Charlson Comorbidity Index, AIDS Acquired Immune Deficiency Syndrome, COPD Chronic Obstructive Pulmonary Disease

**^*^** If participants had a localized and metastatic tumour, they were given a score of 6 in the adjusted CCI.

**Supplementary Table 3**

*Associations between worry and ruminative brooding and subjective and objective physical and cognitive health with anxiety or depression included as an additional covariate*

| ***Subjective Physical Health*** | | | | | | | |
| --- | --- | --- | --- | --- | --- | --- | --- |
| **WHOQoL-Bref** | | | | | | | |
|  | **Worry** (*N=274)* | | | **Ruminative Brooding** (*N=258)* | | |  |
|  | Coefficient (95% CI) | p-value | Adjusted R^2^ | Coefficient (95% CI) | | p-value | Adjusted R^2^ |
| Model | -0.194 (-0.310 to -0.079) | 0.001^**^ | 0.193 | -0.123 (-0.235 to -0.011) | | 0.032^*^ | 0.242 |
| ***Objective Physical Health*** | | | | | | | |
| **CCI**_Adjusted_ | | | | | | | |
|  | **Worry** (*N=252)* | | | **Ruminative Brooding** (*N=236)* | | |  |
|  | Coefficient (95% CI) | p-value | Adjusted R^2^ | Coefficient (95% CI) | p-value | | Adjusted R^2^ |
| Model*^a^* | 0.055 (-0.073 to 0.183) | 0.401 | 0.047 | 0.009 (-0.125 to 0.144) | 0.891 | | 0.044 |
| **FRS**_Adjusted_ | | | | | | | |
|  | **Worry** (*N=274)* | | | **Ruminative Brooding** (*N=258)* | | |  |
|  | Coefficient (95% CI) | p-value | Adjusted R^2^ | Coefficient (95% CI) | | p-value | Adjusted R^2^ |
| Model*^b^* | -0.009 (-0.133 to 0.114) | 0.883 | 0.035 | -0.022 (-0.151 to 0.108) | | 0.745 | 0.024 |
| **SBP** | | | | | | | |
|  | **Worry** (*N=275)* | | | **Ruminative Brooding** (*N=259)* | | |  |
|  | Coefficient (95% CI) | p-value | Adjusted R^2^ | Coefficient (95% CI) | p-value | | Adjusted R^2^ |
| Model | 0.005 (-0.115 to 0.125) | 0.934 | 0.136 | 0.023 (-0.102 to 0.149) | 0.717 | | 0.104 |
| **DBP** | | | | | | | |
|  | **Worry** (*N=274)* | | | **Ruminative Brooding** (*N=258)* | | |  |
|  | Coefficient (95% CI) | p-value | Adjusted R^2^ | Coefficient (95% CI) | p-value | | Adjusted R^2^ |
| Model | -0.010 (-0.136 to 0.117) | 0.883 | 0.029 | 0.036 (-0.093 to 0.166) | 0.584 | | 0.001 |
| ***Subjective Cognitive Difficulties*** | | | |  |  | |  |
| **CDS** |  |  |  |  |  | |  |
|  | **Worry** (*N=275)* |  |  | **Ruminative Brooding** (*N=259)* | | |  |
|  | Coefficient (95% CI) | p-value | Adjusted R^2^ | Coefficient (95% CI) | p-value | | Adjusted R^2^ |
| Model | 0.125 (0.017 to 0.233) | 0.023^*^ | 0.305 | 0.174 (0.063 to 0.284) | 0.002^**^ | | 0.300 |

| ***Objective Cognitive Health*** | |  |  |  |  |  |
| --- | --- | --- | --- | --- | --- | --- |
| **PACC5_Abridged_** | |  |  |  |  |  |
|  | **Worry** (*N=275)* |  |  | **Ruminative Brooding** (*N=259)* | | |
|  | Coefficient (95% CI) | p-value | Adjusted R^2^ | Coefficient (95% CI) | p-value | Adjusted R^2^ |
| Model | -0.030 (-0.138 to 0.077) | 0.578 | 0.298 | -0.081 (-0.198 to 0.035) | 0.174 | 0.242 |

Note. Abbreviations: WHOQoL World Health Organization Quality of Life short version, CCI Charlson Comorbidity Index, FRS Framingham Risk Score, SBP Systolic Blood Pressure, DBP Diastolic Blood Pressure, CDS Cognitive Difficulties Scale, PACC5 Preclinical Alzheimer’s Cognitive Composite 5, N number, CI confidence interval

Model: Adjusted for age, sex, education, cohort, and anxiety (with worry) or depression (with ruminative brooding)

**^a^**CCI_Adjusted_ Model 2 includes only sex, education, and cohort

**^b^**FRS_Adjusted_ Model 2 includes only education and cohort

**Supplementary Table 4**

*Associations between worry and ruminative brooding and subjective and objective physical and cognitive health for the Age-Well and SCD-Well cohorts separately*

| ***Subjective Physical Health*** | | | | | | |
| --- | --- | --- | --- | --- | --- | --- |
| **Worry and WHOQoL-Bref** | | | | | | |
|  | **SCD-Well** (*N=139)* | | | **Age-Well** (*N=135)* | |  |
|  | Coefficient (95% CI) | p-value | Adjusted R^2^ | Coefficient (95% CI) | p-value | Adjusted R^2^ |
| Model 1 | -0.203 (-0.366 to -0.039) | 0.016^*^ | 0.034 | -0.273 (-0.437 to -0.109) | 0.001^**^ | 0.068 |
| Model 2 | -0.238 (-0.401 to -0.075) | 0.005^**^ | 0.098 | -0.278 (-0.440 to -0.116) | 0.001^**^ | 0.102 |
| **Ruminative Brooding and WHOQoL-Bref** | | | | | | |
|  | **SCD-Well** (*N=124)* | | | **Age-Well** (*N=134)* | |  |
|  | Coefficient (95% CI) | p-value | Adjusted R^2^ | Coefficient (95% CI) | p-value | Adjusted R^2^ |
| Model 1 | -0.240 (-0.407 to -0.074) | 0.005^**^ | 0.054 | -0.182 (-0.350 to -0.014) | 0.036^*^ | 0.026 |
| Model 2*^a^* | -0.258 (-0.421 to -0.096) | 0.002^**^ | 0.119 | -0.190 (-0.357 to -0.024) | 0.027^*^ | 0.060 |
| ***Objective Physical Health*** | | | | | | |
| **Worry and CCI_Adjusted_** | | | | | | |
|  | **SCD-Well** (*N=140)* | | | **Age-Well** (*N=112)* | |  |
|  | Coefficient (95% CI) | p-value | Adjusted R^2^ | Coefficient (95% CI) | p-value | Adjusted R^2^ |
| Model 1 | 0.054 (-0.111 to 0.217) | 0.525 | -0.004 | -0.002 (-0.187 to 0.184) | 0.985 | -0.009 |
| Model 2*^b^* | 0.044 (-0.125 to 0.213) | 0.611 | -0.013 | -0.005 (-0.192 to 0.181) | 0.955 | -0.019 |

| **Ruminative Brooding and CCI_Adjusted_** | | | | | | |
| --- | --- | --- | --- | --- | --- | --- |
|  | **SCD-Well** (*N=125)* | | | **Age-Well** (*N=111)* | |  |
|  | Coefficient (95% CI) | p-value | Adjusted R^2^ | Coefficient (95% CI) | p-value | Adjusted R^2^ |
| Model 1 | 0.033 (-0.150 to 0.216) | 0.724 | -0.007 | -0.014 (-0.197 to 0.170) | 0.884 | -0.009 |
| Model 2 | 0.026 (-0.159 to 0.211) | 0.785 | -0.017 | -0.016 (-0.201 to 0.168) | 0.865 | -0.019 |
| **Worry and FRS_Adjusted_** | | | | | | |
|  | **SCD-Well** (*N=141)* | | | **Age-Well** (*N=133)* | |  |
|  | Coefficient (95% CI) | p-value | Adjusted R^2^ | Coefficient (95% CI) | p-value | Adjusted R^2^ |
| Model 1 | 0.106 (-0.057 to 0.269) | 0.206 | 0.004 | 0.050 (-0.070 to 0.171) | 0.416 | -0.001 |
| Model 2 | 0.106 (-0.058 to 0.270) | 0.207 | -0.003 | 0.053 (-0.069 to 0.175) | 0.397 | 0.002 |

**Ruminative Brooding and FRS_Adjusted_**

|  | **SCD-Well** (*N=126)* |  |  | **Age-Well** (*N=132)* |  |  |
| --- | --- | --- | --- | --- | --- | --- |
|  | Coefficient (95% CI) | p-value | Adjusted R^2^ | Coefficient (95% CI) | p-value | Adjusted R^2^ |
| Model 1 | 0.088 (-0.090 to 0.265) | 0.334 | -0.0004 | -0.035 (-0.205 to 0.136) | 0.692 | -0.006 |
| Model 2 | 0.088 (-0.090 to 0.267) | 0.335 | -0.009 | -0.048 (-0.214 to 0.117) | 0.569 | 0.058 |
| **Worry and SBP** | | | | | | |
|  | **SCD-Well** (*N=141)* |  |  | **Age-Well** (*N=134)* |  |  |
|  | Coefficient (95% CI) | p-value | Adjusted R^2^ | Coefficient (95% CI) | p-value | Adjusted R^2^ |
| Model 1 | 0.024 (-0.144 to 0.191) | 0.783 | -0.007 | -0.019 (-0.146 to 0.184) | 0.828 | -0.007 |
| Model 2 | 0.090 (-0.076 to 0.255) | 0.291 | 0.074 | 0.019 (-0.440 to -0.116) | 0.821 | 0.087 |
| **Ruminative Brooding and SBP** | | | | | | |
|  | **SCD-Well** (*N=126)* |  |  | **Age-Well** (*N=133)* |  |  |
|  | Coefficient (95% CI) | p-value | Adjusted R^2^ | Coefficient (95% CI) | p-value | Adjusted R^2^ |
| Model 1 | 0.043 (-0.139 to 0.224) | 0.645 | -0.006 | -0.024 (-0.194 to 0.147) | 0.787 | -0.007 |
| Model 2 | 0.070 (-0.105 to 0.245) | 0.436 | 0.078 | 0.0003 (-0.163 to 0.164) | 0.997 | 0.093 |
| **Worry and DBP** | | | | | | |
|  | **SCD-Well** (*N=141)* |  |  | **Age-Well** (*N=133)* |  |  |
|  | Coefficient (95% CI) | p-value | Adjusted R^2^ | Coefficient (95% CI) | p-value | Adjusted R^2^ |
| Model 1 | 0.095 (-0.070 to 0.260) | 0.261 | 0.002 | -0.001 (-0.187 to 0.157) | 0.864 | -0.007 |
| Model 2 | 0.084 (-0.084 to 0.251) | 0.329 | 0.023 | -0.017 (-0.192 to 0.159) | 0.853 | -0.020 |
| **Ruminative Brooding and DBP** | | | | | | |
|  | **SCD-Well** (*N=126)* |  |  | **Age-Well** (*N=132)* |  |  |
|  | Coefficient (95% CI) | p-value | Adjusted R^2^ | Coefficient (95% CI) | p-value | Adjusted R^2^ |
| Model 1 | 0.026 (-0.037 to 0.318) | 0.101 | 0.014 | -0.048 (-0.218 to 0.121) | 0.576 | -0.005 |
| Model 2 | 0.134 (-0.039 to 0.308) | 0.132 | 0.015 | -0.052 (-0.224 to 0.119) | 0.551 | -0.016 |
| ***Subjective Cognitive Health*** | | | | | | |
| **Worry and CDS** | | | | | | |
|  | **SCD-Well** (*N=140)* |  |  | **Age-Well** (*N=135)* |  |  |
|  | Coefficient (95% CI) | p-value | Adjusted R^2^ | Coefficient (95% CI) | p-value | Adjusted R^2^ |
| Model 1 | 0.116 (-0.009 to 0.321) | 0.067 | 0.017 | 0.263 (0.099 to 0.426) | 0.002^**^ | 0.062 |
| Model 2 | 0.175 (0.009 to 0.341) | 0.041^*^ | 0.056 | 0.260 (0.095 to 0.424) | 0.002^**^ | 0.075 |

**Ruminative Brooding and CDS**

|  | **SCD-Well** (*N=125)* |  |  | **Age-Well** (*N=134)* |  |  |
| --- | --- | --- | --- | --- | --- | --- |
|  | Coefficient (95% CI) | p-value | Adjusted R^2^ | Coefficient (95% CI) | p-value | Adjusted R^2^ |
| Model 1 | 0.228 (0.053 to 0.404) | 0.012^*^ | 0.043 | 0.298 (0.135 to 0.462) | <0.001^***^ | 0.081 |
| Model 2 | 0.241 (0.067 to 0.415) | 0.008^**^ | 0.067 | 0.293 (0.129 to 0.456) | <0.001^***^ | 0.093 |
| ***Objective Cognitive Health*** | | | | | | |
| **Worry and PACC_Abridged_** | | | | | | |
|  | **SCD-Well** (*N=140)* |  |  | **Age-Well** (*N=135)* |  |  |
|  | Coefficient (95% CI) | p-value | Adjusted R^2^ | Coefficient (95% CI) | p-value | Adjusted R^2^ |
| Model 1 | 0.029 (-0.138 to 0.196) | 0.734 | -0.006 | -0.124 (-0.293 to 0.044) | 0.151 | 0.008 |
| Model 2 | -0.051 (-0.208 to 0.107) | 0.529 | 0.157 | -0.164 (-0.310 to 0.017) | 0.030^*^ | 0.272 |
| **Ruminative Brooding and PACC_Abridged_** | | | | | | |
|  | **SCD-Well** (*N=125)* |  |  | **Age-Well** (*N=134)* |  |  |
|  | Coefficient (95% CI) | p-value | Adjusted R^2^ | Coefficient (95% CI) | p-value | Adjusted R^2^ |
| Model 1 | -0.047 (-0.233 to 0.139) | 0.622 | -0.006 | -0.052 (-0.223 to 0.119) | 0.551 | -0.005 |
| Model 2 | -0.082 (-0.253 to 0.089) | 0.350 | 0.157 | -0.073 (-0.222 to 0.075) | 0.335 | 0.252 |

Note. Abbreviations: WHOQoL-Bref World Health Organization Quality of Life short version, CCI Charlson Comorbidity Index, FRS Framingham Risk Score, SBP Systolic Blood Pressure, DBP Diastolic Blood Pressure, CDS Cognitive Difficulties Scale, PACC Preclinical Alzheimer’s Cognitive Composite, N number, CI confidence interval

Model 1: Unadjusted

Model 2: Adjusted for age, sex, and education

**^a^**CCI_Adjusted_ Model 2 includes only sex and education,

**^b^**FRS_Adjusted_ Model 2 includes only education

**Supplementary Table 5**

*Associations between worry and ruminative brooding and unadjusted objective physical and cognitive health in the Age-Well cohort only*

| ***Objective Physical Health*** | | | | | | | | | | | | | |
| --- | --- | --- | --- | --- | --- | --- | --- | --- | --- | --- | --- | --- | --- |
| **Unadjusted CCI** | | | | | | | | | | | | | |
|  | | **Worry** (*N=135)* | | | | | **Ruminative brooding** (*N=134)* | | | | |  |  |
|  | | *Coefficient (95% CI)* | | *p-value* | | *Adjusted R^2^* | *Coefficient (95% CI)* | | *p-value* | | | *Adjusted R^2^* |  |
| Model 1 | | -0.141 (-0.319 to 0.037) | | 0.121 | | 0.011 | -0.124 (-0.309 to 0.061) | | 0.187 | | | 0.006 |  |
| Model 2*^a^* | | -0.140 (-0.320 to 0.040) | | 0.127 | | -0.002 | -0.122 (-0.301 to 0.065) | | 0.199 | | | -0.007 |  |
| **Unadjusted FRS** | | | |  | |  |  | |  | | |  |  |
|  | | **Worry** (*N=133)* | | | | | **Ruminative brooding** (*N=132)* | | | | |  |  |
|  | | *Coefficient (95% CI)* | | *p-value* | | *Adjusted R^2^* | *Coefficient (95% CI)* | | *p-value* | | | *Adjusted R^2^* |  |
| Model 1 | | -0.015 (-0.196 to 0.165) | | 0.866 | | -0.007 | -0.037 (-0.223 to 0.149) | | 0.692 | | | -0.006 |  |
| Model 2*^b^* | | -0.032 (-0.206 to 0.143) | | 0.718 | | 0.062 | -0.052 (-0.232 to 0.128) | | 0.569 | | | 0.058 |  |
| ***Objective Cognitive Health*** | | | | | | | | | | | | | |
| **Unadjusted PACC5** | | | | | | | | | | | | | |
|  | **Worry** (*N=135)* | | | | | | | **Ruminative brooding** (*N=134)* | | |  |  |  |
|  | *Coefficient (95% CI)* | | *p-value* | | *Adjusted R^2^* | | | *Coefficient (95% CI)* | | *p-value* | *Adjusted R^2^* | | |
| Model 1 | -0.139 (-0.317 to 0.039) | | 0.125 | | 0.010 | | | -0.004 (-0.223 to 0.150) | | 0.699 | -0.006 | | |
| Model 2 | -0.178 (-0.334 to -0.021) | | 0.026^*^ | | 0.256 | | | -0.056 (-0.221 to 0.108) | | 0.500 | 0.229 | | |

*Note.* Abbreviations: CCI Charlson Comorbidity Index, CI confidence interval, N number, PACC5 Preclinical Alzheimer’s Cognitive Composite 5

Model 1: Unadjusted

Model 2: Adjusted for age, sex, and education

**^a^**CCI Model 2 includes only sex and education

**^b^**FRS Model 2 includes only education

**Supplementary Table 6**

*Associations between worry and ruminative brooding, included in the same model, and subjective physical and cognitive health*

| ***Subjective Physical Health*** | | | |
| --- | --- | --- | --- |
| **WHOQoL-Bref** |  | | |
|  | **Model** (*N=258)* | | |
|  | **Coefficient (95% CI)** | **p-value** |  |
| **Worry** | -0.144 (-0.275 to -0.013) | 0.033^*^ |  |
| **Ruminative Brooding** | -0.153 (-0.280 to -0.026) | 0.019^*^ |  |
| ***Subjective Cognitive Difficulties*** | |  |  |
| **CDS** |  |  |  |
|  | **Model** (*N=259)* |  |  |
|  | **Coefficient (95% CI)** | **p-value** |  |
| **Worry** | 0.111 (-0.017 to 0.238) | 0.090 |  |
| **Ruminative Brooding** | 0.185 (0.062 to 0.308) | 0.003^**^ |  |

Note. Abbreviations: WHOQoL-Bref World Health Organization Quality of Life short version, CDS Cognitive Difficulties Scale, N number, CI confidence interval

Model: Adjusted for age, sex, education, and cohort

**Supplementary Table 7**

*Associations between demographic variables, worry and ruminative brooding, and subjective and objective physical health*

|  | | | | | | | |
| --- | --- | --- | --- | --- | --- | --- | --- |
| **Subjective Physical Health** | | | | | | | |
| **WHOQoL-Bref** | | | | | | | |
|  | **Worry** (*N=274)* | | | **Ruminative brooding** (*N=258)* | | |  |
|  | *Coefficient (95% CI)* | *p-value* | *Adjusted R^2^* | *Coefficient (95% CI)* | | *p-value* | *Adjusted R^2^* |
| Model A | - | - | 0.115 | - | | - | 0.118 |
| Model B | -0.245 (-0.357 to -0.133) | <0.001^***^ | 0.169 | -0.224 (-0.334 to -0.113) | | <0.001^***^ | 0.167 |
|  | | | | | | | |
| **Objective Physical Health** | | | | | | | |
| **CCI**_Adjusted_ | | | | | | | |
|  | **Worry** (*N=252)* | | | **Ruminative brooding** (*N=236)* | | |  |
|  | *Coefficient (95% CI)* | *p-value* | *Adjusted R^2^* | *Coefficient (95% CI)* | *p-value* | | *Adjusted R^2^* |
| Model A | - | - | 0.044 | - | - | | 0.052 |
| Model B*^a^* | 0.024 (-0.098 to 0.147) | 0.696 | 0.041 | 0.010 (-0.116 to 0.137) | 0.875 | | 0.048 |
| **FRS**_Adjusted_ | | | | | | | |
|  | **Worry** (*N=274)* | | | **Ruminative brooding** (*N=258)* | | |  |
|  | *Coefficient (95% CI)* | *p-value* | *Adjusted R^2^* | *Coefficient (95% CI)* | | *p-value* | *Adjusted R^2^* |
| Model A | - | - | 0.007 | - | | - | 0.008 |
| Model B*^b^* | 0.048 (-0.071 to 0.168) | 0.428 | 0.006 | 0.031 (-0.092 to 0.155) | | 0.617 | 0.005 |
| **SBP** | | | | | | | |
|  | **Worry** (*N=275)* | | | **Ruminative brooding** (*N=259)* | | |  |
|  | *Coefficient (95% CI)* | *p-value* | *Adjusted R^2^* | *Coefficient (95% CI)* | *p-value* | | *Adjusted R^2^* |
| Model A | - | - | 0.108 | - | - | | 0.106 |
| Model B | 0.061 (-0.056 to 0.177) | 0.308 | 0.108 | 0.042 (-0.076 to 0.161) | 0.482 | | 0.104 |
| **DBP** | | | | | | | |
|  | **Worry** (*N=274)* | | | **Ruminative brooding** (*N=258)* | | |  |
|  | *Coefficient (95% CI)* | *p-value* | *Adjusted R^2^* | *Coefficient (95% CI)* | *p-value* | | *Adjusted R^2^* |
| Model A | - | - | 0.010 | - | - | | 0.004 |
| Model B | 0.039 (-0.083 to 0.161) | 0.529 | 0.008 | 0.053 (-0.069 to 0.175) | 0.397 | | 0.002 |

*Note.* Abbreviations: CCI Charlson Comorbidity Index, CI confidence interval, DBP Diastolic Blood Pressure, FRS Framingham Risk Score, N number, SBP Systolic Blood Pressure, WHOQoL World Health Organization Quality of Life short version

Model A: Adjusted for age, sex, education, and cohort

Model B: Adjusted for age, sex, education, and cohort with worry / ruminative brooding included

**^a^**CCI_Adjusted_ Models includes only sex, education, and cohort

**^b^**FRS_Adjusted_ Models includes only education and cohort

**Supplementary Table 8**

*Associations between demographic variables, worry and ruminative brooding, and subjective cognitive difficulties and objective cognitive health.*

|  | | | | | | | | |
| --- | --- | --- | --- | --- | --- | --- | --- | --- |
| **Subjective Cognitive Difficulties** | | | | | | | | |
| **CDS** | | | | | | | | |
|  | **Worry** (*N=275)* | | | **Ruminative brooding** (*N=259)* | | | |  |
|  | *Coefficient (95% CI)* | *p-value* | *Adjusted R^2^* | *Coefficient (95% CI)* | | *p-value* | | *Adjusted R^2^* |
| Model A | - | - | 0.224 | - | | - | | 0.216 |
| Model B | 0.196 (0.091 to 0.302) | <0.001^***^ | 0.258 | 0.239 (0.133 to 0.346) | | <0.001^***^ | | 0.269 |
|  | | | | | | | | |
| **Objective Cognitive Health** | | | | | | | | |
| **PACC5_Abridged_** | | | | | | | | |
|  | **Worry** (*N=275)* | | | | **Ruminative brooding** (*N=259)* | | |  |
|  | *Coefficient (95% CI)* | *p-value* | *Adjusted R^2^* | | *Coefficient (95% CI)* | | *p-value* | *Adjusted R^2^* |
| Model A | - | - | 0.249 | | - | | - | 0.242 |
| Model B | -0.098 (-0.204 to 0.008) | 0.071 | 0.255 | | -0.079 (-0.188 to 0.031) | | 0.160 | 0.245 |

*Note.* Abbreviations: CDS Cognitive Difficulties Scale, CI confidence interval, N number, PACC5 Preclinical Alzheimer’s Cognitive Composite 5

Model A: Adjusted for age, sex, education, and cohort

Model B: Adjusted for age, sex, education, and cohort with worry / ruminative brooding included
